# Template-Free Fabrication of Refractive Index Tunable Polysiloxane Coating Using Homogeneous Embedding Strategy: Application in High-Power Laser System

**DOI:** 10.3390/nano10020381

**Published:** 2020-02-22

**Authors:** Xue-Ran Deng, Xiang-Yang Lei, Wei Yang, Hao-Hao Hui, Tian-Yu Wang, Jin-Ju Chen, Ji-Liang Zhu, Qing-Hua Zhang

**Affiliations:** 1 Research Center of Laser Fusion, China Academy of Engineering Physics, Mianyang 621900, China; xrdeng@foxmail.com (X.-R.D.); leixiangyang2@163.com (X.-Y.L.); hch890@163.com (W.Y.); dream2001hui@163.com (H.-H.H.); wty931121@163.com (T.-Y.W.); 2 School of Materials and Energy, University of Electronic Science and Technology of China, Chengdu 610054, China; jinjuchen@uestc.edu.cn; 3 College of Materials Science and Engineering, Sichuan University, Chengdu 610041, China; jlzhu167@scu.edu.cn

**Keywords:** Tunable refractive index, Homogeneous embedding, Network-sphere structure, High-power laser system

## Abstract

A refractive index (RI) tunable polysiloxane coating was fabricated based on the cross-linked network structure embedded with mesoporous silica nanoparticles (MSNs), in which the MSNs were utilized to modulate the RI as well as to support the interior structure of the polysiloxane coating. The Si–O–Si inorganic backbone structure in combination with characteristics from the photopolymerization of active bonds produced the main cross-linked network structure, and controllable embedding of MSNs constructed the network-sphere structure. This approach eliminated the high-temperature post-treatment that was needed to remove the template, which ensures the safe application for temperature-sensitive laser crystal substrates and avoids coating structure collapse. In addition, degradation of the resulting coating can be minimized due to the similar chemical formation between MSN and polysiloxane coating. Hereby, a polysiloxane coating with expected spectral and laser damage-resistant properties can be obtained. This will facilitate the fabrication and application of a laser component with both high-transmission and high-flux capability for a high-power laser system.

## 1. Introduction

Silica-based coatings are widely applied in the fields of photovoltaics [1,2], LEDs [3], lasers [4,5], energy and fuel [6,7], catalysts [8], and corrosion protection [9], owing to its excellent optical and mechanical properties. In addition, the extraordinary laser damage resistance of silica-based coatings makes it very popular in high-power laser systems as an antireflective (AR) coating [10,11,12], in which precise matching of the refractive index (RI) and thickness for each layer is fundamental to achieve ultrahigh transmission at desired wavelengths. Polysiloxane coatings are one of the silica-based coatings that have been applied in high-power laser systems as the moisture-resistant layer to protect the water-soluble laser crystal KH_2_PO_4_ (KDP) and KD_2_PO_4_ (DKDP) from water invasion [13]. This type of coating is conventionally fabricated through the hydrolysis and polycondensation of siloxane using the sol-gel technique [14,15,16]. However, the RI of such coating is fixed and cannot meet the requirement of KDP/DKDP (these two materials have similar RIs) substrate to achieve ultrahigh transmission at desired wavelengths [13]. Thus, RI modulation of the polysiloxane coating is needed to extend its application in a high-power laser system as both the moisture-resistant and RI-matching layer.

Using a template is a common method to modulate RI by inducing pore structures into a material after removal of the template. RI can be adjusted while the air fills into these pores, and extremely low RI materials have been procured based on the template method [17,18,19,20]. However, heating is the normally used approach to remove the template, and it is a challenge for the temperature-sensitive laser crystals. Moreover, the removal of template may cause lack of supporting for the coating structure and lead to structure deformation or even collapse if lack of supporting. Mixing materials with different RIs is a kind of template-free method to realize RI modulation [21,22,23,24,25], in which nanoparticle filling and organics blending are considered the preferred techniques. Unfortunately, most of the reported filling is based on heterogeneous materials by adding inorganic nanoparticles into oligomer sol, which will induce degradation as well as unexpected light absorption. On the other hand, the modulation range of RI is strongly limited by mixing two or more organics together.

In this paper, a homogeneous embedding method is proposed to modulate the RI of a polysiloxane coating by placing mesoporous silica nanoparticles (MSNs) into the UV-cured polysiloxane coating. The MSN is organic–inorganic with a main Si–O–Si structure and plenty of hydroxyls over its surface, which could help MSNs to incorporate well with the polysiloxane oligomer. Based on this design, the RI of the polysiloxane coating can be continuously modulated in a wide range, and the coating structure can be well protected for further application in high-power laser systems.

## 2. Materials and Methods

### 2.1. Materials

Tetraethylorthosilicate (TEOS) was purchased from Sinopharm Chemical Reagent Co., Ltd. (Beijing, China). Methacryloxypropyltrimethoxysilane (MPS), dipropyleneglycoldiacrylate (DPGDA), 2,4,6-trimethylbenzoyldiphenyl phosphine oxide (TPO), and butanol were purchased from Shanghai Aladdin Bio-Chem Technology Co., Ltd. (Shanghai, China). Anhydrous ethanol, decane, hydrochloric acid, and ammonia water were purchased from Chengdu Kelong Chemical Co., Ltd. (Chengdu, China). The water was deionized. The TEOS and MPS were distillation purified. Other chemicals were used as-purchased.

### 2.2. Synthesis of UV-Curable Polysiloxane Sol

Hydrolysis and polycondensation of MPS were performed under the addition of 1 mol equiv acid water (HCl 0.1 M), corresponding to the stoichiometric proportion. Solvent substitution was applied by the evaporation of produced methanol and further addition of a certain amount of fresh butanol, to reduce the viscosity of the synthesized sol and adjust the concentration of the matrix to ~5wt.%. Decane (1/3 of butanol in volume) in combination with DPGDA (1/10 of butanol in volume) were introduced to modulate the volatilization of this sol and provide excess C=C groups, respectively. The resulting sol was stirred for another 2 h and filtered through a 0.2 µm polyvinylidene fluoride (PVDF) membrane. Then, TPO with a proportion of 1.5wt.% of the total sol was added and stirred before application.

### 2.3. Preparation of Mesoporous Silica Nanoparticles

The colloidal MSNs sol was prepared using TEOS as the matrix, which was mixed with an anhydrous ethanol and ammonium hydroxide aqueous solution (containing 30% ammonia) with stirring at 6 °C for 3 h. The resulting sol was kept still in a sealed glass container for three to five days at room temperature to implement the aging process. It was then refluxed for another 10 h to remove the excess ammonia. This colloidal suspension contained about 3.3% MSNs by weight in ethanol and was filtered through a 0.2 µm PVDF membrane prior to use.

### 2.4. Coating Preparation

The polysiloxane sol and colloidal MSNs sol were mixed in mass proportions of 1:0, 1:4, 1:7, 1:16, and 1:25 to construct the network-sphere structure, in order to tune the coating RI according to different proportions. The mixed sols were spin-coated on the silicon substrates with varied rotation rates and then experienced 100 s of UV irradiation with an intensity of ~200 mW/cm^2^ in order to cure the coatings and keep their thickness nearly the same. After the confirmation of desired coating RI and thickness, a polysiloxane coating accompanied with a specific AR coating was prepared over the KDP/DKDP laser crystals for further verification of spectral and laser damage resistance properties.

### 2.5. Sample Characterizations

The hydrolysis and polycondensation processes of siloxane were characterized using ^29^Si liquid NMR (Bruker AV II-600, Chengdu, China). Viscosity of the sol was measured by a viscometer (BROOKFIELD DV-II+ Pro, Chengdu, China). Nanoindentation (HYSTRON TI 950 TriboIndenter, Chengdu, China) was employed to measure the mechanical properties of the cured coating. The size and distribution property of the MSNs was characterized via TEM (JEM-2500SE, Chengdu, China). FIB-SEM (FEI Helios Nanolab 600i, Chengdu, China) in association with AFM (Bruker Dimension FastScan Pro, Chengdu, China) was utilized to analyze the morphology of the cured coating, especially for the cross-section structure. Microstructure information of the MSNs was analyzed using the dynamic BET method based on nitrogen adsorption–desorption measurement (Micromeritics ASAP 2020 Plus HD88, Chengdu, China). The RI and thickness of the cured coatings were evaluated using ellipsometry (SOPRA GES-5E, Chengdu, China). The reflective spectra of the coating system for application in a high-power laser system were recorded via UV–Vis–NIR spectrometer (PE Lambda 950, Sichuan, China) in the range from 300 to 1100 nm. The laser-induced damage threshold (LIDT) of such polysiloxane coating at desired wavelengths (the beam diameter is about 650 µm, repetition frequency is 1 Hz, and measurement error is within 7% at 1053 nm, and the beam diameter is about 560 µm, repetition frequency is 1 Hz, and measurement error is within 10% at 527 nm) was estimated using R-on-1 mode (the laser was focused on one spot, and its energy continuously increased with each pulse until the coating was broken), in which at least 100 sampling spots were tested in each sample.

## 3. Results and Discussion

### 3.1. Structure Design

**Figure 1:**
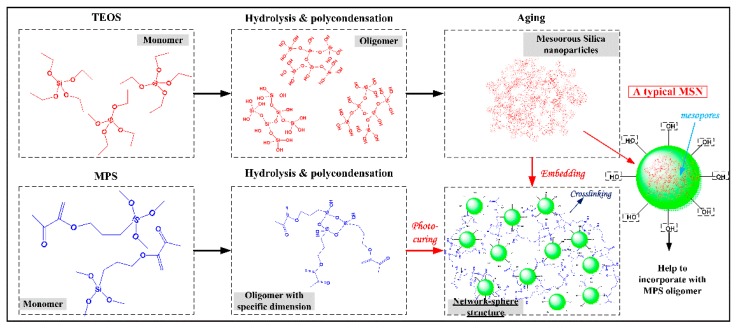
Schematic diagram of the designed network-sphere structure.

A schematic diagram of the network-sphere structure is depicted in Figure 1, in which the cross-linked network structure of the UV-cured polysiloxane coating and uniform embedding of MSNs was designed. Unlike the inorganic nanoparticles, the MSNs applied in this work were synthesized via the hydrolysis approach using TEOS as the matrix and formed by self-assembly of condensed Si–O–Si chains, which endowed these MSNs with numerous residual hydroxyls over the surface and mesopores in the interior. These special characteristics not only provide extra porosity for the embedded coating to modulate the RI, but they also help the MSNs to incorporate well with the hydroxyl-contained MPS oligomer after embedding.

### 3.2. Materials Characterizations

**Figure 2:**
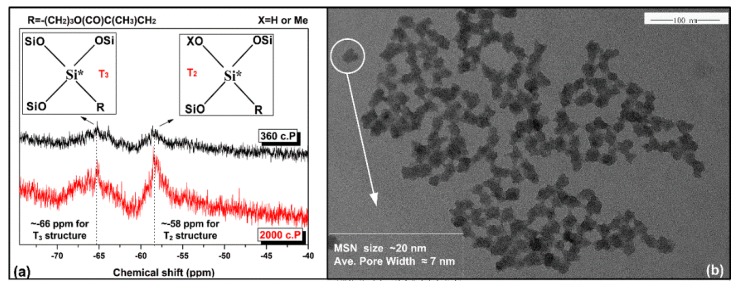
Structure properties of the polysiloxane sol and mesoporous silica nanoparticles (MSNs), (**a**) ^29^Si liquid NMR spectra of polysiloxane sol with different polymerization degrees, (**b**) TEM image of MSNs with information from N_2_ adsorption–desorption analysis.

The structure properties of the synthesized polysiloxane sol were characterized using ^29^Si liquid NMR, and those of MSNs were analyzed via N_2_ adsorption–desorption and TEM. Figure 2a provides the structural characteristics of Si atoms from polycondensed MPS siloxane with varied polymerization degrees. The resulting sols with viscosities of 360 and 2000 c.P were respectively synthesized for 3 and 8 h to implement the polycondensation process. Both spectra consisted of two peaks that were located around −58 ppm (T_2_) and −66 ppm (T_3_), indicating the Si atoms in both sols were connected with 2 or 3 –OSi bonds [26,27]. The peak intensity and area of the polysiloxane sol with a viscosity of 2000 c.P were apparently higher than that of the polysiloxane sol with a viscosity of 360 c.P, implying that longer synthesis time could lead to a higher polymerization degree. A sol with a high polymerization degree could facilitate solidification of a coating and construction of cross-linked structure; thus, a sol with a viscosity of 2000 c.P was considered suitable to fabricate the polysiloxane coating, but proper control in the synthesis process is necessary to avoid unexpected gelation of the sol.

The TEM image in association with N_2_ adsorption–desorption analysis results of the MSNs is shown in Figure 2b. The size of a typical MSN was about 20 nm, and the average pore width was around 7 nm, indicating effective production of inner mesopores from the MSN (the isotherm curves and specific surface area data are respectively given in Figure S1 and Table S1). Moreover, the size of the MSN was proper for embedding, since the thickness of coating in this study was around 100 nm. If the size of the MSN is too large, it cannot be evenly distributed or completely contained inside the coating, which will deteriorate the surface of the embedded coating.

### 3.3. Coating Characterizations

**Figure 3:**
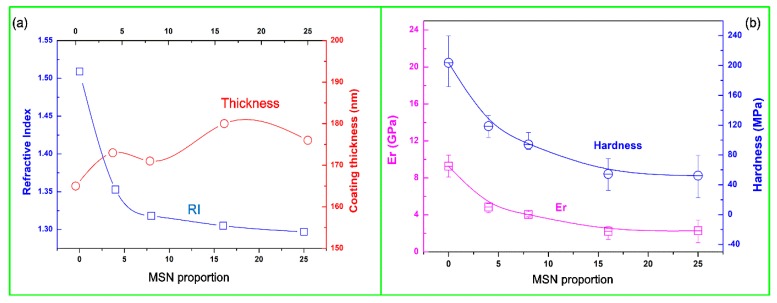
Characterizations of polysiloxane coatings with various MSN embedding proportions, (**a**) RI and thickness results from ellipsometry, (**b**) hardness and elastic modulus Er results from nanoindentation.

The polysiloxane coating was spin-coated using the MSN-embedded polysiloxane sol (the FTIR spectra of the polysiloxane coating before and after curing are given as Figure S2) and then UV-cured, and its RI and thickness with various MSN proportions are illustrated in Figure 3a. The RI coating could be tuned from 1.51 (the intrinsic RI of cured MPS-polysiloxane coating) to 1.30, while the mass proportion of MSN increased from 0 to 25 (the proportion of MPS sol was fixed at 1), indicating the realization of predicted embedding of MSNs and introduction of mesopores inside the coating. Also, the RI decreased dramatically at the beginning of MSN embedding, but it became stable along the further increasing concentration of MSN. This implies a saturation limit for the embedding of MSN even if its concentration keeps increasing. Thus, modulation of the RI coating should be within a proper range.

The mechanical properties of the embedded polysiloxane coatings were characterized using a nanoindenter at the indentation depth about 13 nm (1/10 of the original coating thickness). Coating hardness and elastic modulus Er results are depicted in Figure 3b, in which the same variation trend was observed. Both of these two features decreased with the increase of MSN proportion, indicating that reduction of the cross-linked network structure degraded the mechanical property of the coating. Although the embedding of MSNs was supposed to support the backbone structure, it still could not be compared with the well-formed network structure. This also implies that the mechanical properties of porous materials produced by the removal of internal template would suffer severe degradation since no support was provided inside the coating.

**Figure 4:**
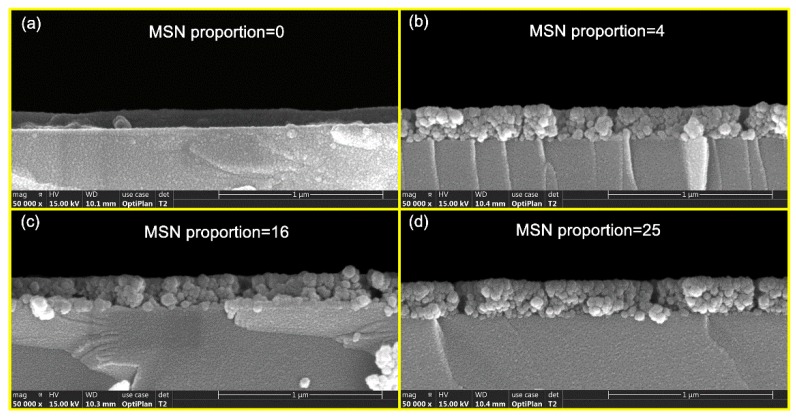
Cross-sectional SEM images of polysiloxane coatings with different MSN embedding proportions, (**a**) with MSN proportion of 0, (**b**) with MSN proportion of 4, (**c**) with MSN proportion of 16, (**d**) with MSN proportion of 25.

Figure 4 demonstrates the cross-section morphology of cured coatings with different MSN embedding ratios, and the hypothesis proposed according to the RI results was verified by the MSN density. No nanoparticles appeared in the pure polysiloxane coating (Figure 4a), and clear embedded nanoparticles can be observed in the coating with an MSN proportion of 4 (Figure 4b). However, enhancement of embedding density could hardly be distinguished (Figure 4c,d) from the SEM images, while the MSN proportion further increased to 16 and 25, which confirms the hypothesis of a saturated MSN embedding limit.

**Figure 5:**
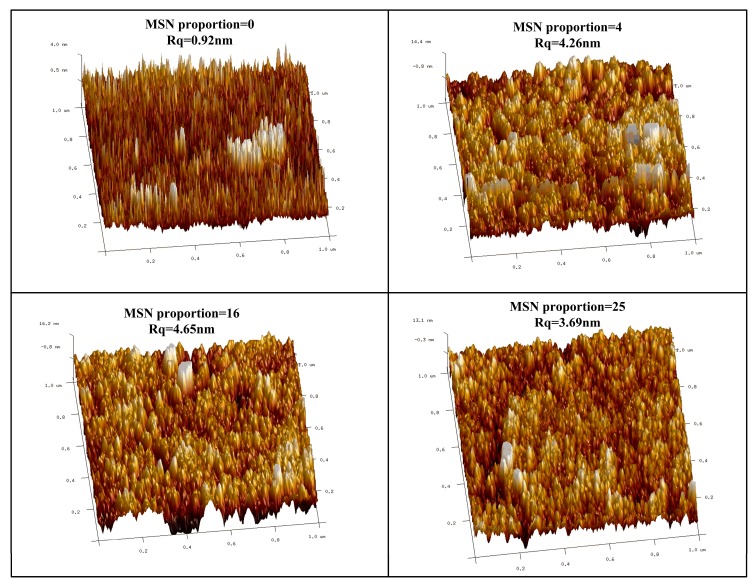
AFM images of polysiloxane coatings with varied MSN embedding proportions.

The surface morphology of polysiloxane coatings was surveyed by AFM, and the results are shown in Figure 5. A smooth coating surface with a Rq of only about 0.92 nm was observed for the pure polysiloxane coating. As MSNs were continuously embedded into the polysiloxane coating, the domain size grew larger and surface roughness deteriorated a little. However, the surface roughness seemed irrelevant to the MSN proportion and kept stable around 4nm for the embedded coatings, indicating that the MSN concentration would not affect the surface morphology under current embedding conditions. This also means complete containing of MSNs inside the polysiloxane coating.

### 3.4. Coating Application

**Figure 6:**
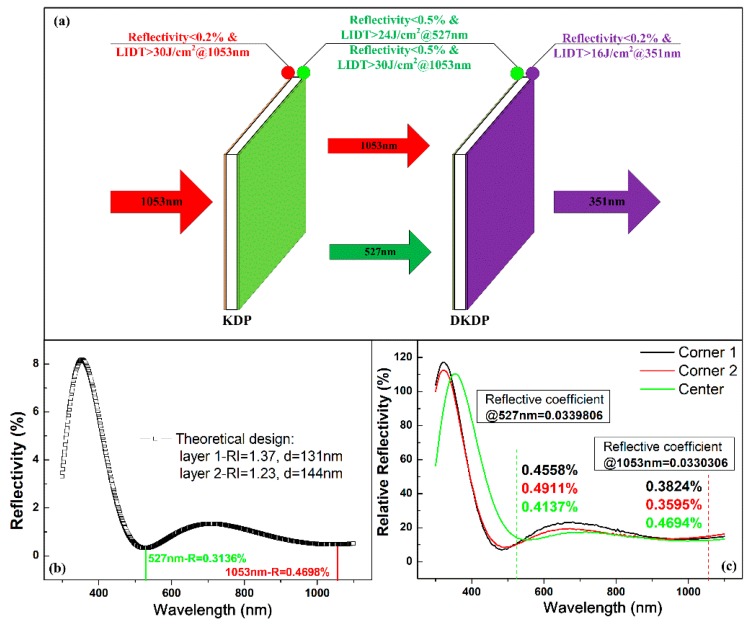
(**a**) Schematic diagram of coating system for nonlinear laser crystals in a high-power laser system, (**b**) spectrum of the theoretically designed coating system, (**c**) spectra of the practically prepared coating system.

A high-power laser system was utilized to drive the controllable fusion according to the theory of inertial confinement fusion [28,29,30], and the incident near-infrared (1053 nm) laser should be converted to a UV (351 nm) laser after passing through the high-power laser system. Nonlinear optical crystals such as KDP and DKDP were employed to implement this conversion duty, and the original 1053 nm laser was first converted to 527 nm (partially) and then converted to 351 nm (shown in Figure 6a) [29,31]. Thus, a coating system that has the capability to enhance the transmission energy both at 1053 and 527 nm was needed. Theoretical calculations from optical design software TFCalc^TM^ [32,33,34] showed that the polysiloxane coating with RI around 1.37 and thickness about 131nm, accompanied with an AR coating whose RI was about 1.23 and thickness around 144nm, could achieve ultrahigh transmission at 1053 nm (>99.5%) and 527 nm (>99.5%), simultaneously (shown in Figure 6b). Optimized from the previous work in this study, a polysiloxane coating with an MSN embedding proportion of 3 was fabricated under the spin velocity of 650 rpm and time of 20 s. The RI and thickness of such coating were measured to be 1.368 and 132 nm after UV curing, respectively. An AR layer formed of silica nanoparticles was also spin-coated and heated at 140 °C for 4 h. Its RI was estimated to be 1.224, and its thickness was tuned to be 147 nm by controlling the spin velocity and time as well. These two layers were combined together as a coating system and prepared sequentially over a 100 mm square KDP substrate, which had a 1° wedged angle, in order to evaluate the residual reflectivity. Spectral testing results are illustrated in Figure 6c (standard fused quartz was first calibrated as the reference, and the corresponding reflective coefficients at 527 and 1053 nm are also given in Figure 6c), in which one point at the center and two points at the corner were measured. Precise matching between theoretical design and practical preparation was observed with the consistent spectral shape, especially for the testing point at the center of the coating system. Not only the spectral shape but also the peak position and reflectivity at target wavelengths were almost the same between the spectrum of the center point and designed one. Although the coating system at the corner blue-shifted a little due to smaller thickness, the reflectivity at the desired wavelength could still meet the ultrahigh requirement. In addition, the spectra at the two corner points showed good consistency, verifying the stability of this coating fabrication technology.

**Figure 7:**
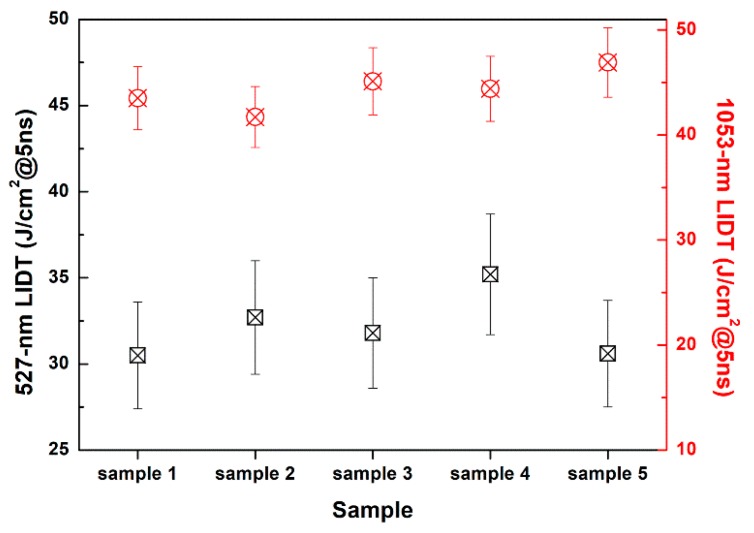
Laser-induced damage threshold (LIDT) results of studied coating system at 527 nm and 1053 nm for five individual samples.

Another crucial feature for the coatings applied in the high-power laser system is the laser damage resistance, which is normally evaluated by LIDT. Generally, this feature is acknowledged to be related with the structure of material, and a porous structure is considered helpful to increase the LIDT because extra space can contribute to buffer and relax the thermal effects produced by the high laser energy [35,36,37]. Therefore, the coating system designed in this study had an advantage since the dense structure of the polysiloxane coating was modified to be porous. As the designed coating system was subjected to laser irradiation at 1053 and 527 nm, LIDT at such wavelengths was characterized under the condition stated previously. Five individual samples were applied for the LIDT test, and the results are demonstrated in Figure 7. The LIDT at 527 nm was above 27 J/cm^2^@5ns, and that at 1053 nm was above 42 J/cm^2^@5ns, confirming the excellent laser damage resistance of this coating system.

## 4. Conclusions

A UV-cured polysiloxane coating with various MSN embedding proportions was fabricated, and the structural, morphological, and mechanical properties have been investigated in this study. The RI coating could be tuned from 1.51 to 1.30 with increasing MSN proportions, but the coating hardness decreased accordingly. In addition, the coating surface morphology was found to be nearly irrelevant to the MSN proportion. MSNs were observed embedded into the polysiloxane until a saturated limit, making excessive addition of MSNs useless for the coating structure. With the MSN proportion of 3, RI of the polysiloxane coating was tuned to be ~1.37 to match that of KDP/DKDP crystal, giving the final coating system ultrahigh transmission above 99.5% at both 527 and 1053 nm. Moreover, the designed porous structure also endowed this polysiloxane coating with excellent LIDT over 27 J/cm^2^@5ns at 527 nm and 42 J/cm^2^@5ns at 1053 nm, thus providing an application option for the high-flux transmission optical component in high-power laser systems.

## Supplementary Materials

The following are available online at https://www.mdpi.com/2079-4991/10/2/381/s1, Figure S1: Nitrogen adsorption–desorption isotherm image of MSNs, Figure S2: FTIR spectra of coating before and after UV curing, Table S1: Specific surface area data of a typical MSN.

## References

[1] Agustin-Saenz C., Machado M., Tercjak A. (2019). Antireflective mesoporous silica coatings by optimization of water content in acid-catalyzed sol-gel method for application in glass covers of concentrated photovoltaic modules. J. Colloid. Interf. Sci..

[2] Aminfard S., Harrison R.K., Ben-Yakar A. (2019). Enhanced optical absorption in ultrathin silicon films using embedded silica-coated silver nanoparticles. Opt. Commun..

[3] Zhang F., Shi Z.F., Ma Z.Z., Li Y., Li S., Wu D., Xu T.T., Li X.J., Shan C.X., Du G.T. (2018). Silica coating enhances the stability of inorganic perovskite nanocrystals for efficient and stable down-conversion in white light-emitting devices. Nanoscale.

[4] Elhadj S., Steele W.A., VanBlarcom D.S., Hawley R.A., Schaffers K.I., Geraghty P. (2017). Scalable process for mitigation of laser-damaged potassium dihydrogen phosphate crystal optic surfaces with removal of damaged antireflective coating. Appl. Opt..

[5] Pan H.W., Kuo L.C., Chang L.A., Chao S., Martin I.W., Steinlechner J., Fletcher M. (2018). Silicon nitride and silica quarter-wave stacks for low-thermal-noise mirror coatings. Phys. Rev. D.

[6] Liu M., Cao M.H., Zeng F.Z., Qi J.L., Liu H.X., Hao H., Zhou Z.H. (2018). Fine-grained silica-coated barium strontium titanate ceramics with high energy storage. Ceram. Int..

[7] Woo J., Yang S.Y., Sa Y.J., Choi W.Y., Lee M.H., Lee H.W., Shin T.J., Kim T.Y., Joo S.H. (2018). Promoting oxygen reduction reaction activity of Fe-N/C electrocatalysts by silica-coating-mediated synthesis for anion-exchange membrane fuel cells. Chem. Mater..

[8] Hamrahian S.A., Rakhtshah J., Davijani S.M.M., Salehzadeh S. (2018). Copper Schiff base complex immobilized on silica-coated Fe_3_O_4_ nanoparticles: A recoverable and efficient catalyst for synthesis of polysubstituted pyrroles. Appl. Organomet. Chem..

[9] Ye Y.W., Liu Z.Y., Liu W., Zhang D.W., Zhao H.C., Wang L.P., Li X.G. (2018). Superhydrophobic oligoaniline-containing electroactive silica coating as pre-process coating for corrosion protection of carbon steel. Chem. Eng. J..

[10] Zhang Q.H., Deng X.R., Yang W., Hui H.H., Wei Y.W., Chen J.J. (2017). Comparative study on cracking behavior of sol-gel silica antireflective coating for high-powered laser system. Eng. Fail. Anal..

[11] Baisden P.A., Atherton L.J., Hawley R.A., Land T.A., Menapace J.A., Miller P.E., Runkel M.J., Spaeth M.L., Stolz C.J., Suratwala T.I. (2016). Large optics for the national ignition facility. Fusion Sci. Technol..

[12] Zhang Q., Wei Y., Yang W., Hui H., Deng X., Wang J., Xu Q., Shen J. (2015). Improvement on contamination resistance to volatile organics and moisture of sol–gel silica antireflective coating for 351 nm laser system by structural modulation with fluorinated compounds. RSC Adv..

[13] Deng X.R., Yang W., Zhang Q.H., Hui H.H., Wei Y.W., Wang J., Xu Q., Lei X.Y., Chen J.J., Zhu J.L. (2018). Fabrication of UV-curable silicone coating with high transmittance and laser-induced damage threshold for high-power laser system. J. Sol.-Gel Sci. Technol..

[14] Elzaabalawy A., Pieter V., Meguid S.A. (2019). Multifunctional silica-silicone nanocomposite with regenerative superhydrophobic capabilities. ACS Appl. Mater. Inter..

[15] Zhang C., Zhang C., Sun J., Ding R., Zhang Q., Xu Y. (2015). A double-layer moisture barrier & antireflective film based on bridged polysilsesquioxane and porous silica. RSC Adv..

[16] Parsaee A., Shokrieh M.M. (2019). Hydrophobic properties of a vulcanized silicone-based nanocomposite coating exposed to heat, sulfuric acid and the ultraviolet radiation. Mater. Res. Express.

[17] Bernsmeier D., Polte J., Ortel E., Krahl T., Kemnitz E., Kraehnert R. (2014). Antireflective coatings with adjustable refractive index and porosity synthesized by Micelle-templated deposition of MgF_2_ sol particles. ACS Appl. Mater. Inter..

[18] Liu C.C., Li J.G., Kuo S.W. (2014). Co-template method provides hierarchical mesoporous silicas with exceptionally ultra-low refractive indices. RSC Adv..

[19] Hsueh H.Y., Chen H.Y., She M.S., Chen C.K., Ho R.M., Gwo S., Hasegawa H., Thomas E.L. (2010). Inorganic gyroid with exceptionally low refractive index from block copolymer templating. Nano. Lett..

[20] Hulkkonen H.H., Salminen T., Niemi T. (2017). Block copolymer patterning for creating porous silicon thin films with tunable refractive indices. ACS Appl. Mater. Inter..

[21] Landwehr J., Fader R., Rumler M., Rommel M., Bauer A.J., Frey L., Simon B., Fodor B., Petrik P., Schiener A. (2014). Optical polymers with tunable refractive index for nanoimprint technologies. Nanotechnology.

[22] Tsai C.L., Liou G.S. (2015). Highly transparent and flexible polyimide/ZrO_2_ nanocomposite optical films with a tunable refractive index and Abbe number. Chem. Commun..

[23] Mohamed-Noriega N., Hinojosa M., Gonzalez V., Rodil S.E. (2016). Polymer-based composite with outstanding mechanically tunable refractive index. Opt. Mater..

[24] Seo Y., Cho S., Kim S., Choi S., Kim H. (2017). Synthesis of refractive index tunable silazane networks for transparent glass fiber reinforced composite. Ceram. Int..

[25] Chen M., Zhang G.Y., Liang X., Zhang W.S., Zhou L., He B.F., Song P., Yuan X., Zhang C.H., Zhang L.Y. (2016). Thermally stable transparent sol-gel based active siloxane-oligomer materials with tunable high refractive index and dual reactive groups. RSC Adv..

[26] Wang S., Chen K., Wang Q. (2018). Ytterbium triflate immobilized on sulfo-functionalized SBA-15 catalyzed conversion of cellulose to lactic acid. J. Porous Mat..

[27] Ataollahi N., Cappelletto E., Vezzu K., Di Noto V., Cavinato G., Callone E., Dire S., Scardi P., Di Maggio R. (2018). Properties of anion exchange membrane based on polyamine: Effect of functionalized silica particles prepared by sol-gel method. Solid State Ionics.

[28] Meyer-ter-Vehn J. (1997). Prospects of inertial confinement fusion. Plasma Phys. Contr. F.

[29] Miller G.H., Moses E.I., Wuest C.R. (2004). The national ignition facility. Opt. Eng..

[30] Moses E.I., Wuest C.R. (2005). The national ignition facility: Laser performance and first experiments. Fusion Sci. Technol..

[31] Deng X.R., Yang W., Hui H.H., Zhang Q.H., Xu Q., Chen J.J., Zhu J.L., Lei X.Y. (2019). Silica-Based Sol-Gel Coating with high transmission at 1053 and 527 nm and absorption at 351 nm for frequency-converting crystals in high-power laser system. Appl. Sci..

[32] Hu D., Liu D., Zhang J., Wu L., Li W. (2018). Preparation and stability study of broadband anti-reflection coatings and application research for CdTe solar cell. Opt. Mater..

[33] Khan S.B., Irfan S., Zheng Z., Lee S.L. (2019). Influence of refractive index on antireflectance efficiency of thin films. Materials.

[34] Ye L., Ge X., Wang X., Hui Z., Zhang Y. (2019). Design and preparation of durable double-layer non-quarter-wave antireflective coatings. Ceram. Int..

[35] Ristau D., Jupe M., Starke K. (2009). Laser damage thresholds of optical coatings. Thin Solid Films.

[36] Krupta R., Giesen A. (1996). Photothermal study of optical components at 10.6 µm: Finite element calculations and experiments. Proc. SPIE.

[37] Zhao Q., Wu Z., Thomsen M., Han Y., Fan Z. (1998). Interfacial effects on the transient temperature rise of multilayer coatings induced by a short-pulse laser irradiation. Proc. SPIE.

