# Supplementary material for: Template-Free Fabrication of Refractive Index Tunable Polysiloxane Coating Using Homogeneous Embedding Strategy: Application in High-Power Laser System
Source: Nanomaterials (Basel). 2020 Feb 22;10(2):381. doi: 10.3390/nano10020381 (PMC7075301; doi:10.3390/nano10020381)
Supplement: Supplementary file 1 [file nanomaterials-10-00381-s001.pdf]

Supplementary materials

Template-Free Fabrication of Refractive Index Tunable Polysiloxane Coating Using Homogeneous Embedding Strategy: Application in High-Power Laser System

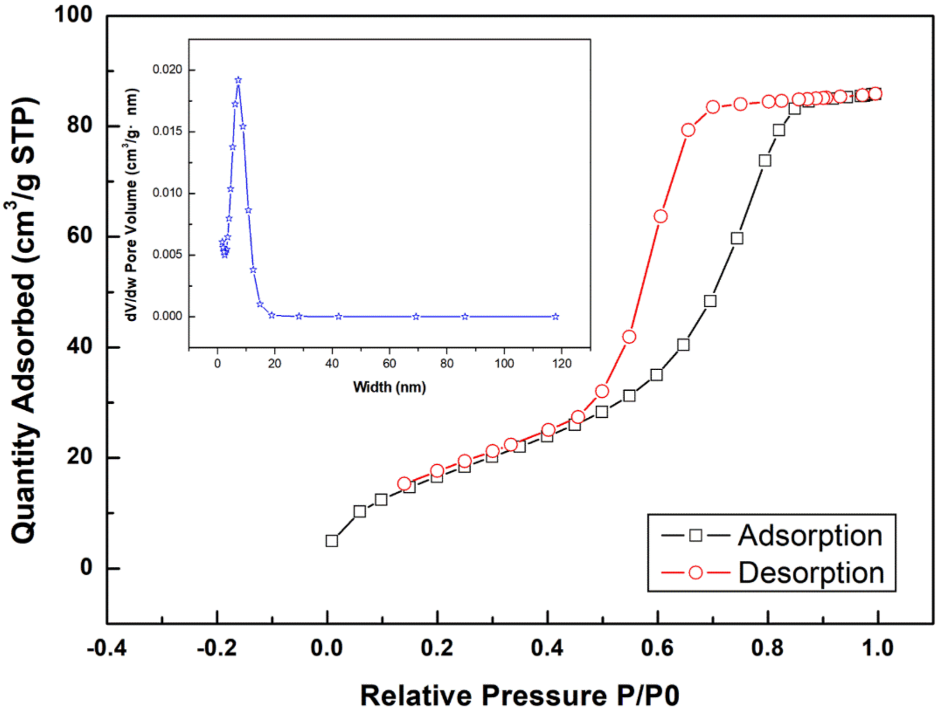

Figure S1. Nitrogen adsorption-desorption isotherm image of MSNs.

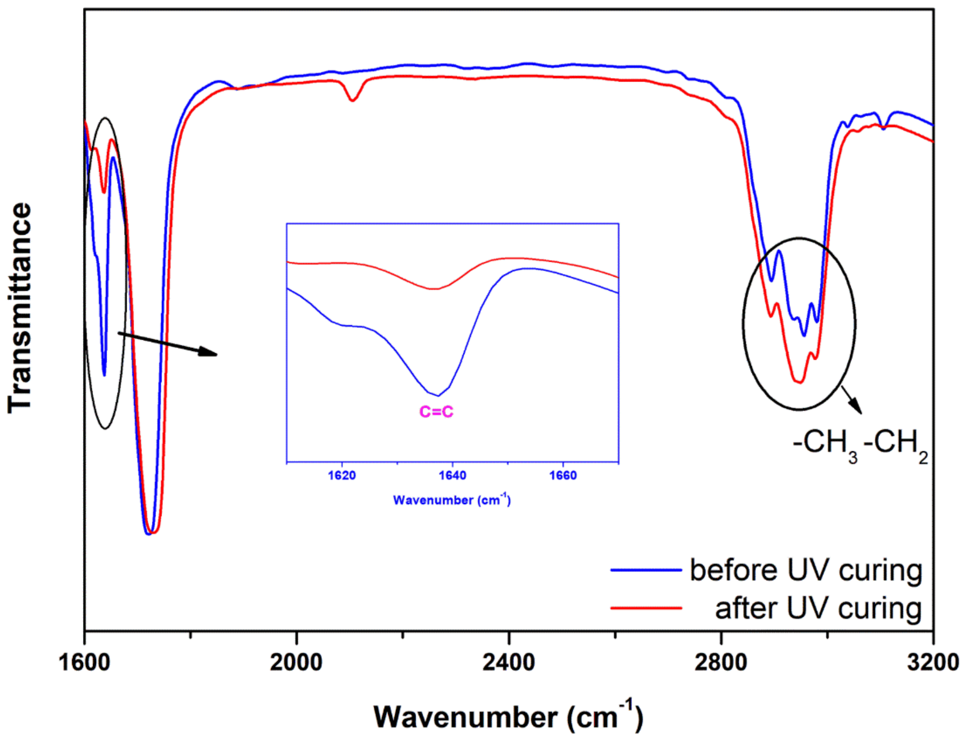

Figure S2. FTIR spectra of coating before and after UV curing.

Table S1. Specific Surface Area data of typical MSN.

**Single point surface area at p/p° = 0.299522363:**	**61.5860 m²/g**
BET Surface Area:	67.8628 m²/g
t-Plot external surface area:	90.8852 m²/g
BJH Adsorption cumulative surface area of pores between 1.7000 nm and 300.0000 nm width:	85.2113 m²/g
BJH Desorption cumulative surface area of pores between 1.7000 nm and 300.0000 nm width:	115.0010 m²/g
